# Epidemiological Situation of Monkeypox Transmission by Possible Sexual Contact: A Systematic Review

**DOI:** 10.3390/tropicalmed7100267

**Published:** 2022-09-27

**Authors:** Darwin A. León-Figueroa, Joshuan J. Barboza, Edwin A. Garcia-Vasquez, D. Katterine Bonilla-Aldana, Milagros Diaz-Torres, Hortencia M. Saldaña-Cumpa, Melissa T. Diaz-Murillo, Olga Campos-Santa Cruz, Alfonso J. Rodriguez-Morales

**Affiliations:** 1 Facultad de Medicina Humana, Universidad de San Martín de Porres, Chiclayo 15011, Peru; 2 Centro de Investigación en Atención Primaria en Salud, Universidad Peruana Cayetano Heredia, Lima 15102, Peru; 3 Sociedad Científica de Estudiantes de Medicina Veritas (SCIEMVE), Chiclayo, Peru; 4 Vicerrectorado de Investigación, Universidad Norbert Wiener, Lima 15046, Peru; 5 Grupo de Investigación Biomedicina, Faculty of Medicine, Fundacion Universitaria Autonoma de Las Americas, Pereira 660001, Risaralda, Colombia; 6 Latin American Network of MOnkeypox VIrus Research (LAMOVI), Pereira, Risaralda, Colombia; 7 Master of Clinical Epidemiology and Biostatistics, Universidad Cientifica del Sur, Lima 15067, Peru

**Keywords:** Monkeypox, sexual contact, orthopoxvirus, sex with men, monkeypox virus

## Abstract

Monkeypox (MPX), a zoonotic infection caused by the monkeypox virus (MPXV), has re-emerged worldwide with numerous confirmed cases with person-to-person transmission through close contacts, including in sexual networks. Therefore, this study aimed to determine the epidemiological situation of monkeypox transmission by possible sexual contact. A systematic literature review was conducted using PubMed, Scopus, Web of Science, and Embase databases until 18 August 2022. The key search terms used were “monkeypox”, “sexual contact”, “sexual intercourse” and “sexual transmission”. A total of 1291 articles were retrieved using the search strategy. After eliminating duplicates (n = 738) and examining by title, abstract, and full text, 28 studies reporting case reports of monkeypox with a detailed description of clinical features, sexually transmitted diseases, method of diagnosis, location and course of skin lesions, and treatment were included. A total of 4222 confirmed cases of monkeypox have been reported, of which 3876 monkeypox cases are the result of transmission by sexual contact distributed in twelve countries: 4152 cases were male with a mean age of 36 years. All confirmed cases of monkeypox were diagnosed by reverse transcriptase-polymerase chain reaction (RT-PCR). The most frequent clinical manifestations were fever, lymphadenopathy, headache, malaise, and painful perianal and genital lesions. The most frequent locations of the lesions were perianal, genital, oral, trunk, upper and lower extremities. Patients were in good clinical condition, with treatment based on analgesics and antipyretics to relieve some symptoms of monkeypox. A high proportion of STIs and frequent anogenital symptoms were found, suggesting transmissibility through local inoculation during close skin-to-skin or mucosal contact during sexual activity. The highest risk of monkeypox transmission occurs in men who have sex with men, and MPXV DNA could be recovered in seminal fluid. It is essential to establish health policies for the early detection and management of patients with monkeypox.

## 1. Introduction

Monkeypox (MPX) has re-emerged on a global scale with numerous cases confirmed across the globe in 2022 [1]. The rapid spread of cases across different countries has raised serious concern among public health officials worldwide, prompting accelerated investigations aimed at identifying the origins and cause of the rapid spread of cases [2,3]. As of 25 August 2022, 46,724 confirmed cases of monkeypox have been reported in 98 countries worldwide [4]. According to the World Health Organization (WHO), the outbreak continues to primarily affect men who have sex with men, who have reported recent sex with one or more male partners [5]. In addition, the most frequently reported and suspected route of transmission among known contacts has been through possible sexual contact.

MPX is a zoonotic viral disease caused by the monkeypox virus (MPXV) [6,7]. MPXV is a double-stranded DNA virus of the genus Orthopoxvirus of the family Poxviridae [8,9], first identified as a human pathogen in the Democratic Republic of Congo (DRC, formerly Zaire) in 1970 [10,11]. MPXV has two distinct genetic clades: the Central African clade (Congo basin) and the West African clade [11,12]. The mortality rate varies between 1% and 10%, depending on the clade, and children, pregnant women, and immunocompromised individuals are at high risk of a fatal outcome [13].

Individuals with MPX have an incubation period of 7 to 21 days before the onset of clinical manifestations [14], such as fever, headache, muscle aches, back pain, chills, rash, and lymphadenopathy [15,16]. Complications of MPX may include pneumonitis, encephalitis, visibly life-threatening keratitis, and secondary bacterial infections [17,18].

MPX is transmitted to humans by direct contact with an infected person or animal or by contact with virus-contaminated material [15,19,20]. The virus is spread by oral and nasopharyngeal fluid exchange or intradermal injection; it replicates rapidly at the site of inoculation and spreads to adjacent lymph nodes [21]. In addition, most of these patients presented atypical skin lesions with lesions in the genital and perianal region [22]. Therefore, it is important to evaluate sexual transmission and to take into consideration patients with human immunodeficiency virus (HIV) and sexually transmitted infections (STI) [23].

Currently, monkeypox has no definitive vaccine or drug; it is treated by controlling symptoms and preventing or ameliorating complications [24]. However, the United States has recommended a licensed vaccine, JYNNEOS (Smallpox and Monkeypox Vaccine, Live, Nonreplicating) for vaccination of persons at risk of occupational exposure to Orthopoxviruses [25].

The objective of the present study is to determine the epidemiological situation of monkeypox transmission by possible sexual contact.

## 2. Materials and Methods

### 2.1. Protocol and Registration

This protocol follows the recommendations established by the Preferred Reporting Items for Systematic Reviews and Meta-Analyses (PRISMA) statement [26], and it has been reported in the International Prospective Register of Systematic Reviews (PROSPERO) database (CRD42022340855).

### 2.2. Eligibility Criteria

To assess the prevalence of sexual contact transmission of monkeypox, we included peer-reviewed published articles with study designs of case reports, case series, and observational studies (cohort and nonrandomized intervention studies). No language limit was established for the articles and publications were included until 18 August 2022. Systematic review articles, narrative reviews, randomized clinical trials, editorials, letters to the editor, and conference proceedings were excluded.

### 2.3. Information Sources and Search Strategy

A systematic search was carried out in PubMed, Scopus, Web of Science and Embase. The search terms used were: (“Monkeypox” OR “Monkey Pox”) AND (“sexual contact” OR “sexual intercourse” OR “sexual behavior” OR “transmission” OR “sexual transmission” OR “Sexual Intercourse” OR “Intercourse, Sexual” OR “Coital” OR “Copulation” OR “Sexual relations”) (Table 1). The searches were completed on 18 August 2022, and four different investigators independently evaluated the search results.

### 2.4. Study Selection

Three investigators (D.A.L.F., E.G.V., J.J.B.) created a database based on the electronic searches, managed with the appropriate management software (EndNote), and duplicates were removed. Then, through Rayyan QCRI [27] three researchers (M.T.D.M., M.D.T. and O.C.S.) carried out the screening process, analyzing the titles and abstracts provided by the search independently, choosing those that appeared to meet the inclusion criteria and, if necessary, evaluating the full text. In case of disagreement, the investigators will discuss until a consensus is reached; in case of dispute, a fourth investigator will be invited to the discussion to help resolve it.

The authors (D.K.B.A. and A.J.R.M.) reviewed the full-text reports and analyzed the inclusion criteria to reach a decision.

### 2.5. Outcomes

The primary outcome was to report the epidemiological situation of monkeypox transmission by possible sexual contact.

### 2.6. Data Collection Process and Data Items

Four investigators independently extracted data from the selected studies into a Microsoft Excel spreadsheet. The following data were extracted from the selected studies: author data, date of publication, study design, country, sex, age, sexual behavior, sexually transmitted infections (STIs), signs and symptoms, diagnostic test, days from systemic symptoms to lesion onset, location of skin lesions, evolution of lesions, and treatment. A fifth investigator checked the list of articles and data extractions to ensure that there were no duplicate articles or duplicate information and resolved discrepancies about study inclusion.

## 3. Results

### 3.1. Study Selection

**Figure 1:**
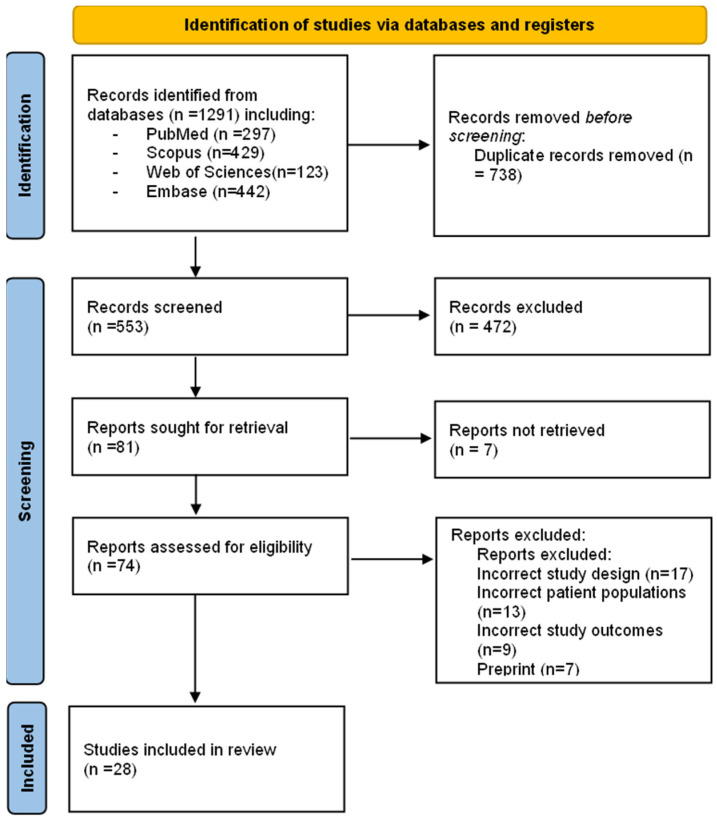
PRISMA flow chart of the studies selection process.

A total of 1291 articles were retrieved using the search strategy. The selection strategy is shown in the prism flow chart (Preferred Reporting Items for Systematic Reviews and Meta-Analyses) [26]. After the removal of duplicates (n = 738), 553 articles were screened by the reviewers. After filtering the titles and reading the abstracts, 74 articles were selected for full-text reading, and 28 were considered eligible for inclusion in this systematic review [7,8,22,28,29,30,31,32,33,34,35,36,37,38,39,40,41,42,43,44,45,46,47,48,49,50,51,52] (Figure 1).

### 3.2. Study Characteristics

The main characteristics of the articles included in this review are summarized in Table 2. Our review included 28 studies that were published between 1 January and 18 August 2022 [7,8,22,28,29,30,31,32,33,34,35,36,37,38,39,40,41,42,43,44,45,46,47,48,49,50,51,52]. The studies (n = 28) reported case reports of sexually transmitted monkeypox with a detailed description of the number of cases, clinical manifestations, history of sexually transmitted diseases, method of diagnosis, location and course of skin lesions, and treatment (Table 2 and Table 3). A total of 4222 confirmed cases of monkeypox were reported [7,8,22,28,29,30,31,32,33,34,35,36,37,38,39,40,41,42,43,44,45,46,47,48,49,50,51,52], of which 3876 cases of monkeypox were the result of sexual contact transmission, distributed in twelve countries: Germany (n = 357) [38,43,48], Korea (n = 1) [39], Spain (n = 1924) [41,42,44,46,51], Italy (n = 42) [7,8,50], United Kingdom (n = 364) [31,33,37,47,52], Australia (n = 1) [32], Nigeria (n = 16) [45], United States (n = 1140) [22,30], Portugal (n = 28) [29,34], France (n = 1) [35], Romania (n = 1) [36], and Czech Republic (n = 1) [28] (Table 1). Spain was the country with the highest number of cases of sexually transmitted monkeypox, followed by the United States and the United Kingdom.

### 3.3. Demographical Characteristics and Diagnostic Method for Monkeypox

Of the total number of cases (n = 4222) registered with monkeypox [7,8,22,28,29,30,31,32,33,34,35,36,37,38,39,40,41,42,43,44,45,46,47,48,49,50,51,52], 4152 cases were found to be male [7,8,22,28,29,30,31,32,33,34,35,36,37,38,39,40,41,42,43,44,45,46,47,48,49,50,51,52]. The average age of reported cases with monkeypox was 36 years. Of the reported cases with monkeypox, 3479 had a sexual behavior of being men who have sex with men [7,8,28,29,30,31,32,34,35,36,37,38,39,40,41,42,43,44,45,46,47,48,49,50,51,52] and 112 cases had a sexual behavior of being gay or bisexual or men who have sex with men [22,33,52]. In addition to the cases reported with monkeypox transmitted by sexual contact: Syphilis (n = 24) [8,28,30,32,37,40,41], Gonorrhea (n = 4) [41,48] and herpes simplex (n = 25) [8,28,30,32,37,48] were the most prevalent sexually transmitted infections and 949 patients tested positive for human immunodeficiency virus [7,8,28,29,31,32,34,35,36,37,38,40,42,44,45,46,47,48,49,50,51,52]. All confirmed cases of monkeypox were diagnosed by Polymerase chain reaction with reverse transcriptase (RT-PCR) [7,8,22,28,29,30,31,32,33,34,35,36,37,38,39,40,41,42,43,44,45,46,47,48,49,50,51,52] (Table 2).

### 3.4. Clinical Manifestations, Localization of Skin Lesions and Treatment

The most frequent clinical manifestations in patients confirmed with monkeypox were fever (n = 1521) [7,8,22,28,29,30,31,32,34,35,36,38,39,40,41,42,44,45,46,47,48,49,51], lymphadenopathy (n = 1385) [7,22,28,29,30,31,34,35,36,37,38,39,40,42], headache (n = 956) [22,31,32,38,39,40,41,42], skin lesions or rash (n = 1925) [28,29,34,36,37,39,42,44,45,46,47,48,49,51], painful perianal and genital lesions (n = 499) [7,28,29,30,31,34,35,36,44,45,46,47,48,49,51] (Table 3). The average number of days from systemic symptoms to the appearance of lesions was 3 (Table 3). The most frequent lesion locations were perianal (n = 1209) [7,8,22,28,30,31,34,36,38,41,42,44,45,46,47,48,49,50,51], genital (n = 1373) [8,22,29,30,31,32,34,36,37,39,40,41,42,44,45,46,47,48,49,50,51], oral (n = 812) [22,31,32,37,38,39,41,44,46,47,48,49,51], trunk (n = 640) [7,8,22,32,36,44,46,47,48,49,51] and upper and lower extremities (n = 491) [7,8,22,29,30,32,36,37,44,46,47,48,49] (Table 3). The evolution of these lesions was asynchronous. Most of the patients did not report a specific treatment, but simply followed their treatments for the sexually transmitted diseases they were suffering from [7,8,28,29,30,31,32,35,36,37,38,39,40,41,42,44,45,46,47,48,49,50,51,52].

## 4. Discussion

Currently, MPX represents the most recent emerging zoonotic disease worldwide [53]. For this reason, the main objective of the present systematic review is to determine the epidemiological situation of monkeypox transmission by possible sexual contact. It is important to have knowledge of the clinical characteristics, sexual behavior, localization and evolution of skin lesions, diagnosis, and correct management of these patients.

This study reported 3876 cases of monkeypox through possible sexual contact transmission distributed in twelve countries. It was found that 85% of the reported cases were from Europe, with Spain being the country with the most reports. All patients were diagnosed by RT-PCR. The majority of patients reported an average age of 36 years and were male. The most recent outbreak of Monkeypox (MPXV) in 2022 has brought new light to the importance of this sexual transmission mechanism in the spread of an emerging pathogen [54,55]. All reported patients had sexual risk behaviors, of which men who have sex with men (MSM) was the most prevalent.

According to the WHO, current epidemiological data show a predominance of the involvement of young males, with 98.2% (20,138/20,500) of cases with available data on gender being male with a median age of 36 years (interquartile range: 30–43 years). Among cases with declared sexual orientation, 95.8% (9484/9899) identified as men who have sex with men. Sexual encounters were the most common type of transmission, accounting for 5954 of 7250 (82.1%) of all transmission cases [56]. In the recently released study by Thornhill JP et al., 528 instances of monkeypox were documented, of which 98% were homosexual or bisexual men who had engaged in risky sexual activity, and 41% had human immunodeficiency virus infection [57].

The incubation period has been estimated at 5 to 21 days and the duration of symptoms and signs at 2 to 5 weeks [58]. The disease begins with nonspecific symptoms and signs, the most frequent symptoms reported in the study cases were fever, lymphadenopathy, headache, malaise, and general lesions. All lesions had an asynchronous evolution, with the genital and anal regions being the most frequent locations. This suggests that contact in sexual intercourse could be a risk factor for transmission [8] because it can occur through contact with infected humans, or with human body material containing the virus [59]; therefore, sexual intercourse without the use of a condom could be another risk factor, since there are other viruses found in semen [60]. However, there are still no studies demonstrating the presence of Monkeypox in this body secretion, except for case reports from Italy and Germany [7,8,51].

To determine a rapid and definitive diagnosis of MPX, the exudate from lesions can provide the best sample [61]. This is performed through direct recognition of viral DNA by real-time PCR, allowing rapid discrimination between smallpox and other poxviruses [61,62,63,64,65]. In addition, it is important to understand that MPXV DNA could be recovered in blood, urine, upper respiratory tract, and seminal fluid [8,16,61].

According to the study by Ranjit Sah et al., monkeypox virus is highly prevalent in seminal samples from monkeypox cases, supporting the idea that the disease is sexually transmitted. However, since the virus can reproduce in this environment, this high prevalence rate does not always suggest viral contagiousness [66]. The infectivity of seminal monkeypox virus remains debatable and requires further investigation.

Sixty-nine percent of the cases presented had a previous STI, the most frequent being syphilis and hepatitis. In addition, most of them were HIV positive, which led us to infer that this history could be a risk factor that may contribute to infection [54]. MPX can be confused with some sexually transmitted infections (STIs) that can cause skin rashes, for example, syphilis, human immunodeficiency virus (HIV), chancroid, condyloma acuminate, disseminated gonorrhea, and herpes [67].

Most of the patients had symptomatic treatment, although some did not require any specific treatment. Recently, some drugs were developed in the United States to treat smallpox infection. These antiviral agents are also active against MPXV. The Food and Drug Administration (FDA) approved tecovirimat in 2018, which acts by inhibiting the viral protein p27, thus preventing viral egress from infected cells, and oral brincidofovir in 2021, which blocks viral DNA polymerase [68].

It is important to follow up on the contacts of the reported cases to avoid the spread of this disease, taking into account the number of days from the general symptoms to the appearance of lesions, which ranged from 1 to 5 days in the cases reported. The study did not report any deaths in cases of monkeypox potentially transmitted by sexual contact, although this also depends on the immunological status of the patient and associated complications.

The situation of this new zoonotic disease, which now appears to be emerging as an STI, is of great concern and warrants further study to understand the multiple effects of this virus, which is currently affecting several continents and with possible new routes of transmission, including during the COVID-19 pandemic that has not yet ended [69].

## 5. Conclusions

The reemerging zoonotic disease (monkeypox) has spread rapidly throughout the world and has shown unusual reports of person-to-person transmission through possible sexual contact. The prevalence of STIs and the frequent occurrence of anogenital symptoms point to local inoculation during intimate skin-to-skin or mucosal contact during sexual activity. Men who have sex with men are most at risk of spreading monkeypox, and MPXV DNA can be found in seminal fluid. The establishment of health policies is crucial for the early identification and treatment of people with monkeypox.

## Figures and Tables

**Table 1:** Bibliographic search strategy.

Base	Search Strategy
PUBMED	#1 (“Monkeypox” OR “Monkey Pox”) #2 (“sexual contact” OR “sexual intercourse” OR “sexual behavior” OR “transmission” OR “sexual transmission” OR “Sexual Intercourse” OR “Intercourse, Sexual” OR Coital OR Copulation OR “Sexual relations”) #3 = #1 AND #2
SCOPUS	#1 TITLE-ABS-KEY (“Monkeypox” OR “Monkey Pox”) #2 TITLE-ABS-KEY (“sexual contact” OR “sexual intercourse” OR “sexual behavior” OR “transmission” OR “sexual transmission” OR “Sexual Intercourse” OR “Intercourse, Sexual” OR Coital OR Copulation OR “Sexual relations”) #3 = #1 AND #2
WEB OF SCIENCE	#1 ALL = (“Monkeypox” OR “Monkey Pox”) #2 ALL = (“sexual contact” OR “sexual intercourse” OR “sexual behavior” OR “transmission” OR “sexual transmission” OR “Sexual Intercourse” OR “Intercourse, Sexual” OR Coital OR Copulation OR “Sexual relations”) #3 = #1 AND #2
EMBASE	#1 ‘monkeypox’/exp OR ‘monkeypox’ OR ‘monkeypox virus’/exp OR ‘monkeypox virus’ #2 ‘sexual contact’ OR ‘sexual behavior’ OR transmission OR ‘sexual transmission’ OR ‘sexual intercourse’ OR ‘intercourse, sexual’ OR coital OR copulation OR ‘sexual relations’ #3 = #1 AND #2

**Table 2:** Characteristics of included studies and description of case reports of monkeypox.

Authors	Year	Design	Country	Number of Cases (N)	Cases by Sexual Contact (N)	Age (Years)	Sex (M/F)	Sexual Behavior	Previous STIs	HIV	Recent Sexual Exposure	Diagnostic Method for Monkeypox
**Antinori, A. et al.** [8]	2022	Case reports	Italy	4	1	Median: 30	M	MSM	Hepatitis C, syphilis	Positive	Yes	RT-PCR
					2		M	MSM	Syphilis	Negative	Yes	RT-PCR
					3		M	MSM	Syphilis, hepatitis B	Positive	Yes	RT-PCR
					4		M	MSM	Hepatitis A	Negative	Yes	RT-PCR
**Heskin, J. et al.** [31]	2022	Case reports	United Kingdom	2	1	NR	M	MSM	None	Negative	Yes	RT-PCR
					2	NR	M	MSM	None	Positive	Yes	RT-PCR
**Hammerschlag, Y. et al.** [32]	2022	Case report	Australia	1	1	30	M	MSM	Syphilis	Positive	Yes	RT-PCR
**Minhaj, F.S. et al.** [22]	2022	Case reports	United States	17	16	Median 40 (28–61)	NR	GBMSM	NR	NR	Yes	RT-PCR
**Vivancos, R. et al.** [33]	2022	Case reports	United Kingdom	86	66	Median: 38 (32–43)	M (79/79)	GBMSM (66/79)	NR	NR	Yes	RT-PCR
**Perez Duque, M. et al.** [34]	2022	Case reports	Portugal	27	27	Median: 33 (22–51)	M	MSM (18/19), MSW (1/19)	NR	Positive (n = 14)	Yes	RT-PCR
**Vallée, A. et al.** [35]	2022	Case report	France	1	1	NR	M	MSM	HIV	Positive	Yes	RT-PCR
**Oprea, C. et al.** [36]	2022	Case report	Romania	1	1	26	M	MSM	HIV	Positive	Yes	RT-PCR
**Bížová, B. et al.** [28]	2022	Case report	Czech Republic	1	1	34	M	MSM	Syphilis	Positive	Yes	RT-PCR
**Patrocinio-Jesus, R. et al.** [29]	2022	Case report	Portugal	1	1	31	M	MSM	HIV	Positive	Yes	RT-PCR
**Basgoz, S.N. et al.** [30]	2022	Case report	United States	1	1	31	M	MSM	Syphilis, herpes simplex	Negative	Yes	RT-PCR
**Mileto, D. et al.** [7]	2022	Case report	Italy	1	1	33	M	MSM	HIV	Positive	Yes	RT-PCR
**Girometti, N. et al.** [37]	2022	Cohort study	United Kingdom	54	54	Median: 41 (34–45)	M	MSM	HIV (n = 13) syphilis (n = 14), herpes simplex (n = 24) and gonorrhea (n = 13)	Positive	Yes	RT-PCR
**Noe, S. et al.** [38]	2022	Case report	Germany	2	1	26	M	MSM	HIV	Positive	Yes	RT-PCR
					2	32	M	MSM	NR	NR	NR	RT-PCR
**Jang, Y.R. et al.** [39]	2022	Case report	Korea	1	1	34	M	MSM	None	NR	NR	RT-PCR
**Maronese, C.A. et al.** [40]	2022	Case report	Italy	1	1	44	M	MSM	Hepatitis C, HIV, syphilis	Positive	Yes	RT-PCR
**Peiró-Mestres, A. et al.** [41]	2022	Case report	Spain	12	1	30	M	MSM	None	Positive (n = 4)	Yes	RT-PCR
					2	30	M	MSM	Syphilis		Yes	RT-PCR
					3	40	M	MSM	None		Yes	RT-PCR
					4	40	M	MSM	None		Yes	RT-PCR
					5	40	M	MSM	None		Yes	RT-PCR
					6	30	M	MSM	None		Yes	RT-PCR
					7	40	M	MSM	None		Yes	RT-PCR
					8	50	M	MSM	Syphilis		Yes	RT-PCR
					9	40	M	MSM	None		Yes	RT-PCR
					10	30	M	MSM	None		Yes	RT-PCR
					11	30	M	MSM	None		Yes	RT-PCR
					12	30	M	MSM	Chlamydia y gonorrhea		Yes	RT-PCR
**Iñigo Martínez, J. et al.** [42]	2022	Case report	Spain	508	427	Median: 35 (18–67)	M (n = 503) F (n = 5)	MSM (n = 397)	NR	Positive (n = 225)	Yes	RT-PCR
**Selb, R. et al.** [43]	2022	Case report	Germany	521	349	Median: 38 (32–44)	M	MSM (n = 349)	NR	NR	Yes	RT-PCR
**Tarín-Vicente, E.J. et al.** [44]	2022	Cohort study	Spain	181	181	Median: 37 (31–42)	M (n = 175) F (n = 6)	MSM (n = 166) MSW (n = 15)	HIV (n = 72)	Positive	Yes	RT-PCR
**Ogoina, D. et al.** [45]	2022	Cross-sectional study	Nigeria	16	16	Median: 28 (22–43)	M (n = 12)F (n = 4)	MSW (n = 16)	HIV (n = 3)	Positive (n = 3)	Yes	RT-PCR
**Orviz, E. et al.** [46]	2022	Observational study	Spain	48	48	Median: 35 (29–44)	M	MSM (n = 42)	HIV (n = 19)	Positive (n = 19)	Yes	RT-PCR
**Patel, A. et al.** [47]	2022	Case report	United Kingdom	197	197	Median: 38 (32–42)	M	MSM	HIV (n = 70)	Positive (n = 70)	Yes	RT-PCR
**Pfäfflin, F. et al.** [48]	2022	Case report	Germany	1	1	Range (41–50)	M	MSM	None	Positive	Yes	RT-PCR
				2	2	Range (21–30)	M	MSM	None	Negative	Yes	RT-PCR
				3	3	Range (31–40)	M	MSM	None	Negative	Yes	RT-PCR
				4	4	Range (31–40)	M	MSM	Syphilis (blood), gonorrhea (rectal)	Negative	Yes	RT-PCR
				5	5	Range (21–30)	M	MSM	Gonorrhea, Ureaplasma, Mycoplasma hominis (all urethral)	Negative	Yes	RT-PCR
				6	6	Range (31–40)	M	MSM	Gonorrhea (rectal)	Positive	Yes	RT-PCR
**Philpott, D. et al.** [49]	2022	Case report	United States	1195	1123	Median: 35 (30–41)	M (n = 1178) F (n = 5)	MSM	HIV (n = 490)	Positive (n = 490)	Yes	RT-PCR
**Raccagni, A.R. et al.** [50]	2022	Case report	Italy	36	36	Median: 41.5 (31.25–35.5)	M	MSM	HIV (n = 15)	Positive (n = 15)	Yes	RT-PCR
**Rodríguez, B.S. et al.** [51]	2022	Case report	Spain	1256	1256	Median: 37	M (n = 1242) F (n = 14)	MSM	NR	NR	Yes	RT-PCR
**Vusirikala, A. et al.** [52]	2022	Case report	United Kingdom	45	45	Median: 37	M	GBMSM	HIV (n = 11)	Positive (n = 11)	Yes	RT-PCR

MSM:  men who have sex with men; MSW: men who have sex with women; GBMSM: gay or bisexual or other men who have sex with men; STI: sexually transmitted infection; HIV: human immunodeficiency virus; RT-PCR: Polymerase chain reaction with reverse transcriptase; M/F: Male/Female; NR:   No report.

**Table 3:** Characteristics of eligible studies. Clinical manifestations, localization, the evolution of lesions, and treatment of monkeypox cases.

Authors	Number of Cases (N)	Symptoms and Findings in Physical Examination	Days from Systemic Symptoms to Appearance of Lesion	Localization of Skin Lesions	Evolution of Lesions	Treatment
**Antinori, A. et al.** [8]	1	No	NR	Genital, thorax and calf area.	Asynchronous	Ciprofloxacin, acyclovir, and benzylpenicillin
	2	Fever	3	Anal, back, legs and foot sole.	Asynchronous	NR
	3	Fever	3	Anal, head, thorax, legs, arms, hand, and genital area.	Asynchronous	anti-inflammatories and antihistamines
	4	Myalgia	2	Genital and pubic area.	Asynchronous	NR
**Heskin, J. et al.** [31]	1	Lymphadenopathy, fever, headache, and diarrhea. Perioral white patches and painful lesions with perianal blisters.	1	Perioral and perianal.	Asynchronous	Intravenous ceftriaxone
	2	Lymphadenopathy, fever, headache, and diarrhea. Perioral papules, papules on the mons pubis and penile shaft that evolved into painful ulcers.	2	Genital, pubic and tongue, oral and buccal mucous membranes.	Asynchronous	Intravenous ceftriaxone, antibiotic therapy.
**Hammerschlag, Y. et al.** [32]	1	Fever and general malaise	3	Penis, trunk, face, extremities, hand, calf, nasal throat.	Asynchronous	Intramuscular ceftriaxone, oral doxycycline, oral cephalexin, intravenous cephalorin and oral analgesia.
**Minhaj, F.S. et al.** [22]	17	Rash (n = 17), Fatigue or malaise (n = 13), Chills (n = 12), Lymphadenopathy (n = 9), Headache (n = 8), Fever (n = 7), Body aches (n = 6), Sore throat or cough (n = 5), Sweat (n = 4).	NR	Arm (n = 9), Trunk (n = 9), Leg (n = 8), Face (n = 7), Hand (n = 6), Perianal (n = 6), Oral (n = 5), Neck (n = 5), Genital (penis or vagina) (n = 4), Feet (n = 4).	Asynchronous	NR
**Vivancos, R. et al.** [33]	86	NR	NR	NR	NR	NR
**Perez Duque, M. et al.** [34]	27	Exanthema (n = 14), inguinal lymphadenopathy (n = 14), fever (n = 13), genital ulcers (n = 6)	NR	Anus (n = 14) and genitalia (n = 12)	Asynchronous	NR
**Vallée, A. et al.** [35]	1	Fever, severe fatigue, chills, myalgia, sore throat, severe anal pain, and lymphadenopathy.	5	None	Asynchronous	No specific treatment.
**Oprea, C. et al.** [36]	1	High fever (up to 39 degrees Celsius), chills, rectal pain, vesiculopustular rash, dysphagia, severe pain in the anorectal region, marked hyperemia of the pharynx, with pseudomembranous appearance, and palatal petechiae, aphthous ulcers, lymphadenopathy.	4	Anogenital, buttocks, neck, trunk, upper and lower limbs, and sole of one foot.	Asynchronous	Symptomatic, fluid, and topical treatment for aphthous ulcers and pharyngeal hyperemia.
**Bížová, B. et al.** [28]	1	High fever, chills, lymphadenopathy, rash, painless perianal erosions, and perianal umbilicated papules.	3	The perianal and left side of the body	Asynchronous	Antibiotic therapy
**Patrocinio-Jesus, R. et al.** [29]	1	Painless genital rash, fever, sore throat, macular rash, lymphadenopathy.	2	Genitals and hands	Asynchronous	No specific intervention
**Basgoz, N. et al.** [30]	1	Rectal pain, vesiculopustular rash, rectal bleeding, foul-smelling and mucopurulent discharge, fever, chills, lymphadenopathy, and swelling in the groin.	3	Perianal, penis, arms, and legs.	Asynchronous	Penicillin G benzathine, ceftriaxone, valacyclovir, doxycycline, and intravenous acyclovir.
**Mileto, D. et al.** [7]	1	Asthenia, fever, general malaise, anorexia, papular lesions on both elbows, ulcerated perianal lesion, pharyngodynia, bilateral inguinal lymphadenopathy.	3	Perianal, face, both elbows, trunk, buttock, and right foot.	Asynchronous	Dolutegravir, rilpivirine, isolated in a negative pressure room.
**Girometti, N. et al.** [37]	54	Fatigue (n = 36), fever (n = 31), myalgia (n = 16), sore throat (n = 11), lymphadenopathy (n = 30) and skin lesions (n = 54).	3	Skin (n = 54), genitalia (n = 33), perianal (n = 24), upper and lower extremities (n = 27), facial (n = 11), oropharyngeal (n = 4) and torso (n = 14).	Asynchronous	No specific treatment was recorded and all individuals improved clinically.
**Noe, S. et al.** [38]	1	General malaise, fever, arthralgia, myalgia and back pain, headache, dysphagia, and presence of white spots on his tonsils.	2	Tonsils, trunk, limbs, and head.	Asynchronous	No specific treatment was recorded.
	2	Fever, fatigue, cough, inguinal lymphadenopathy, and anal pain.	2	Trunk	Asynchronous	No specific treatment was recorded.
**Jang, Y.R. et al.** [39]	1	Headache, fever, rash, lymphadenopathy, and chills.	3	Penis, oropharynx, nasopharynx, face, abdomen, and trunk.	Asynchronous	No specific treatment was recorded.
**Maronese, C.A. et al.** [40]	1	Fever, headache, malaise, and lymphadenopathy.	5	Penis, scrotum, and extremities.	Asynchronous	No specific treatment was recorded.
**Peiró-Mestres, A. et al.** [41]	1	Myalgia, fatigue	NR	Arm, perianal area and trunk	Asynchronous	No specific treatment was recorded.
	2	Odynophagia, general malaise		Genital area	Asynchronous	No specific treatment was recorded.
	3	Myalgia, fever, Proctitis		Anal area	Asynchronous	No specific treatment was recorded.
	4	Proctalgia, odynophagia, general malaise		Perianal, chest and trunk	Asynchronous	No specific treatment was recorded.
	5	Fever, myalgia, general malaise		Chest and legs	Asynchronous	No specific treatment was recorded.
	6	Fever, proctitis		Wrist, pectoral, fingers, hand and perianal area	Asynchronous	No specific treatment was recorded.
	7	Headache, general malaise		Ulcerated ventral tongue	Asynchronous	No specific treatment was recorded.
	8	General malaise, fever		Trunk and genital area	Asynchronous	No specific treatment was recorded.
	9	Myalgia, general malaise		Genital lesions	Asynchronous	No specific treatment was recorded.
	10	General malaise, myalgia, proctitis		Perianal area	Asynchronous	No specific treatment was recorded.
	11	NR		Genital area	Asynchronous	No specific treatment was recorded.
	12	Myalgia, general malaise		Genital and anal area	Asynchronous	No specific treatment was recorded.
**Iñigo Martínez, J. et al.** [42]	508	Exanthema (n = 498), fever (n = 324), lymphadenopathy (n = 311), asthenia (n = 238), myalgia (n = 185), headache (n = 162), odynophagia (n = 143), and proctitis (n = 81)	NR	Anogenital and/or perineal area (n = 359), legs and/or arms (n = 222), face (n = 177), chest and/or abdomen (n = 159), back (n = 132), palms and/or plants (n = 124).	Asynchronous	No specific treatment was recorded.
**Selb, R. et al.** [43]	521	NR	NR	NR	NR	NR
**Tarín-Vicente, E.J. et al.** [44]	181	Influenza-like illness (n = 147), Fever (n = 131), Headache (n = 96), Sore throat (n = 66) and lymphadenopathy (n = 153)	NR	Genital (n = 100), Perianal (n = 66), Oral ulcer (n = 45), Perioral (n = 51), Hands and feet (n = 108), Trunk and extremities (n = 104)	Asynchronous	No specific treatment was recorded.
**Ogoina, D. et al.** [45]	16	Fever (n = 9), Genital rash (n = 4), facial rash (n = 3)	NR	Genital (n = 13)	Asynchronous	No specific treatment was recorded.
**Orviz, E. et al.** [46]	48	Fever (n = 25), Asthenia (n = 32), Myalgia (n = 25), Inguinal lymphadenopathies (n = 30), Other location of lymphadenopathies (n = 9), Headache (n = 25), Proctitis (n = 13), Urethritis (n = 7), Rash (n = 4), Nasal congestion (n = 4), and Cough (n = 8)	NR	Vesicular-umbilicated skin lesions location (n = 45), Genitals (n = 26), Upper extremities (n = 20), Perianal (n = 17), Trunk (n = 16), Facial (n = 12), Periorally (n = 9), Lower extremities (n = 10), and Palms and soles (n = 2)	Asynchronous	No specific treatment was recorded.
**Patel, A. et al.** [47]	197	Mucocutaneous manifestations (n = 197), Fever (n = 122), Headache (n = 49), Fatigue/lethargy (n = 46), Myalgia (n = 62), Arthralgia (n = 21), Back pain (n = 21), Rectal pain or pain on defecation (n = 71), and Lymphadenopathy (n = 114)	NR	Face (n = 71), Trunk (n = 70), Arms/legs (n = 74), Hands/feet (n = 56), Genitals (n = 111), Anus or perianal area (n = 82), and Oropharyngeal (n = 27)	Asynchronous	No specific treatment was recorded.
**Pfäfflin, F. et al.** [48]	1	Fever, Perianal pain, Anal abscess, and Lymphadenopathy	NR	Limbs	Asynchronous	Ibuprofen
	2	Fever, malaise, anal pain, and anal fissure	NR	Left arm	Asynchronous	Metamizole, tramadol, lidocaine
	3	Anal pain, Rectal ulcer, and proctitis	NR	Limbs	Asynchronous	Ibuprofen, metamizole, lidocaine
	4	Fatigue, Anal pain, and Anal ulcer	NR	Arms, trunk, genital	Asynchronous	Metamizole, lidocaine, Penicillin G benzathine, ceftriaxone
	5	Fever, malaise, myalgia, sweats, Anal pain, Inflammation of sigmoid, rectum and anal canal	NR	Head, neck, trunk, limbs	Asynchronous	Metamizole, lidocaine, Ceftriaxone, azithromycin
	6	Myalgia, fever, malaise, Anal pain, Anal ulcer, proctitis	NR	Legs	Asynchronous	Metamizole, lidocaine, Ceftriaxone, azithromycin
**Philpott, D. et al.** [49]	1195	Rash (n = 1004), Fever (n = 596), Chills (n = 550), Lymphadenopathy (n = 545), Malaise (n = 531), Myalgia (n = 507), Headache (n = 469), Rectal pain (n = 201), Pus or blood in stools (n = 184), Abdominal pain (n = 96), Rectal bleeding (n = 90), Tenesmus (n = 90), and vomiting or nausea (n = 83)	NR	Genitals (n = 333), Arms (n = 284), Face (n = 276), Legs (n = 265), Perianal (n = 225), Mouth, lips, or oral mucosa (n = 179), Palms of hands (n = 157), Trunk (n = 156), Neck (n = 130), Head (n = 97), and Soles of feet (n = 77)	Asynchronous	No specific treatment was recorded.
**Raccagni, A.R. et al.** [50]	36	NR	NR	Genital (n = 13), Rectal (n = 18), cutaneous (n = 20)	Asynchronous	No specific treatment was recorded.
**Rodríguez, B.S. et al.** [51]	1256	Report of some cases (n = 530): Fever (n = 302), lymphadenopathy (n = 216), Asthenia (n = 224), Muscle pain (n = 167), Throat pain (n = 136), and Headache (n = 140)	NR	Report of some cases (n = 530): Anogenital (n = 355), other than anogenital or oro/peribuccal (n = 293)	Asynchronous	No specific treatment was recorded.
**Vusirikala, A. et al.** [52]	45	NR	NR	NR	Asynchronous	No specific treatment was recorded.

NR:  No report.

## Data Availability

This section provides details regarding where data supporting reported results can be found, including links to publicly archived datasets analyzed or generated during the study.
